# Two New AChE Inhibitors Isolated from Li Folk Herb Heilaohu “*Kadsura coccinea*” Stems

**DOI:** 10.3390/molecules24193628

**Published:** 2019-10-08

**Authors:** Sheng Zhuo Huang, Lin Ping Duan, Hao Wang, Wen Li Mei, Hao Fu Dai

**Affiliations:** 1 Hainan Key Laboratory for Research and Development of Natural Products from Li Folk Medicine, Institute of Tropical Bioscience and Biotechnology, Chinese Academy of Tropical Agriculture Sciences, Haikou 571101, China; huangshengzhuo@itbb.org.cn (S.Z.H.); wanghao@itbb.org.cn (H.W.); 2 College of Food Science and technology, Nanjing Agricultural University, Nanjing 210095, China; 15854846579@163.com

**Keywords:** *Kadsura coccinea*, *Schisandraceae*, AChE inhibitor, triterpenoid

## Abstract

Two new triterpenoids, named kadsuricoccins A and B, together with three known ones, were isolated from the Li folk herb Heilaohu, the stems of *Kadsura coccinea* (Lem.) A. C. Smith, which was used for food and as a healthy supplement. Their structures were elucidated by comprehensive analyses of mass spectrometry (MS) and nuclear magnetic resonance (NMR) spectroscopic data. To search healthy components, an acetylcholinesterase (AChE) inhibitory activity test by Ellman’s Method was conducted, kadsuricoccins A and B showed activity with the AChE inhibit index (AII) up to 68.96% ± 0.19% and 57.8% ± 0.11% at 94 nM (compared with positive control tacrine AII 79.80% ± 0.20%, 9.4 nM), respectively.

## 1. Introduction

**Figure 1:**
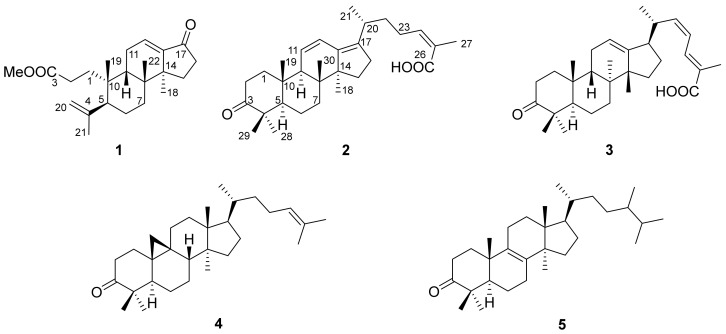
The structures of compounds **1**–**5**.

With global exchange increase and dietary habit diversification, ethnic characteristic foods are becoming more and more popular. One of the unique ingredients of ethnic characteristic foods is their flavor agent, which could stimulate appetite, protect foods from spoilage and keep people healthy [1]. Living in the dangerous rainforest, the Li folk ancestors in Hainan Island had tested and screened out various herbs to treat disease and to improve their quality of life [2]. Among them, the Heilaohu [*Kadsura coccinea* (Lem.) A. C. Smith, *Schisandraceae*] fruit is a new taste, and its stems with their full aroma and sedative effect is used for pork and beef stew by the Li folk people [3]. The chemical constituents studied of this plant showed unique features in previous studies, and series of lignans [4], triterpenoids [5] and nor-terpenoids [6], with biological activities, were isolated [4,7]. To reveal the mechanism of action and explore new agents for health care, we studied *K. coccinea* stems (collected from the Li folk area) and found two new compounds, named kadsuricoccins A and B, and three known compounds: (22*Z*,24*E*)-3-oxoprotosta-27 12,22,24-trien-26-oic acid (**3**) [8], cycloartenone (**4**) [9] and 24-methyl-8-lanoten-3-one (**5**) [10] (Figure 1). We then tested their AChE inhibit activities to search for natural material in preventing Alzheimer’s Disease (AD). The isolation process, new compounds’ structural elucidation details and the AChE inhibit assay, were described in this paper.

## 2. Results and Discussion

**Figure 2:**
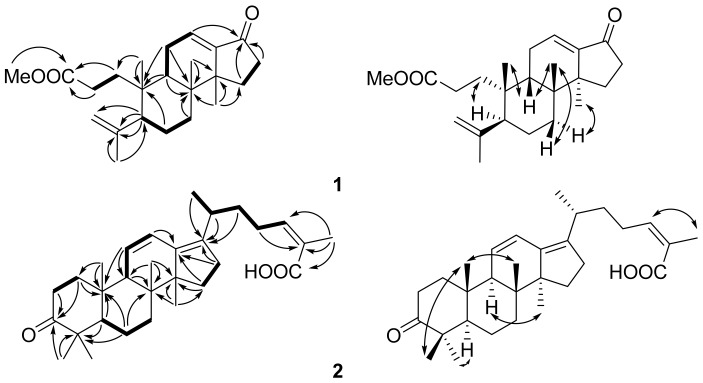
Key ^1^H-^1^H COSY (**▬**), HMBC (H→C), and ROESY (↔) correlations of **1**–**2**.

Kadsuricoccins A (**1**) was isolated as colorless, amorphous powder. Its molecular formula of C_23_H_34_O_3_ was determined by the positive HRESIMS ion at *m*/*z* 381.2508 [M + Na]^+^ (calcd. for C_23_H_34_O_5_Na, 381.2508), indicative of seven degrees of unsaturation. The IR spectrum revealed the presence of carbonyl (1,712 and 1,691 cm^−1^) and double bonds (1,623 and 1,344 cm^−1^) absorptions. The ^1^H-NMR spectra (Table 1) of compound **1** exhibited signals of one methoxyl [*δ*_H_ 3.69 (3H, s, -OMe)], four methyls [*δ*_H_ 1.18 (3H, s, H-18), 0.90 (3H, s, H-19), 1.82 (3H, s, H-21) and 0.94(3H, s, H-22)], and three olefinic protons (*δ*_H_ 4.96 (1H, s, Ha-20), 4.84 (1H, s, Hb-20) and 6.65 (1H, dd, *J* = 3.4, 3.6 Hz, H-12). The ^13^C-NMR and DEPT spectroscopic data (Table 1) showed 23 carbon resonances, including five methyls (one methoxyl), eight methylenes (one olefinic), three methines (one olefinic) and seven quaternary carbons (two carbonyl and two olefinic). By comparing the NMR, compound **1** was similar to those of 16-oxo-mansumbin-3(28),13(17)-dien-3-oic-acid methyl ester [11], a rare octanordammarane (a kind of octanary nor-triterpenoid) except for the markedly different shifts at *δ*_C_ 131.4 (d, C-12), 144.1 (s, C-13), 36.1 (t, C-16) and 207.1 (s, C-17), instead of *δ*_C_ 27.3 (t, C-12), 187.0 (s, C-13), 209.0 (s, C-16) and 126.5 (s, C-17) in 16-oxo-mansumbin-3(28),13(17)-dien-3-oic-acid methyl ester, indicating that compound **1** was generated from 16-oxo-mansumbin-3(28),13(17)-dien-3-oic-acid methyl ester for the α,β- unsaturated lactone moved from C-13-C-17-C(=O)-16 to C-12-C-13-C(=O)-17. Furthermore, the determined molecular formula C_15_H_22_O_4_ and the key ^1^H-^1^H COSY correlations with H-11 [*δ*_H_ 2.21 (2H, m)]/H-12, H-15[*δ*_H_ 1.99 (1H, m) and 1.54 (1.54)]/H-16 [*δ*_H_ 2.46 (1H, m) and 2.28 (1H, m)], and the HMBC correlations from H-12 to C-17 (*δ*_C_ 27.3 t) and C-10 (*δ*_C_ 37.7 s), and from H-15 and H-16 to C-17 verified this hypothesis. The other ^1^H-^1^HCOSY and HMBC correlations (Figure 2) also confirmed this atom connectivity. The relative configuration of compound **1** was determined to be likely to the 16-oxo-mansumbin-3(28),13(17)-dien-3-oic-acid methyl ester (Figure 2) with β-oriented of C-19, H-10 [11], and C-22, and *α*-oriented of H-5 and C-18 by ROESY cross-peaks H-2 [*δ*_H_ 2.29 (1H, m)]/H-5 [*δ*_H_ 2.18 (1H, m)], H-19/H-9 [*δ*_H_ 2.25 (1H, m)], H-9/H-22, H-22/H-7β [*δ*_H_ 2.08 (1H, m)], and H-7α [*δ*_H_ 1.17 (1H, m)]/H-18. Thus, the structure of compound **1** was assigned as shown, and named kadsuricoccins A (**1**).

Kadsuricoccins B (**2**)’s molecular formula of C_30_H_44_O_3_ was determined by the positive HRESIMS ion at *m*/*z* 475.3183 [M + Na]^+^ (calcd. for C_23_H_34_O_5_Na, 381.2508) with nine degrees of unsaturation. The IR spectrum showed the presence of carbonyl (1705 cm^−1^) and double bonds (1457 and 1380 cm^−1^) absorptions. The ^13^C-NMR and DEPT spectroscopic data (Table 1) revealed 30 carbon resonances, including seven methyls, eight methylenes, six methines (three olefinic), and nine quaternary carbons (one ketone, one acidic, and three olefinic). By comparing the NMR, compound **2** was similar to those of kadindutic acid [12], except for the markedly different shifts at *δ*_C_ 37.5 (s, C-8), 46.9 (d, C-9), 121.5 (d, C-11), 126.6 (d, C-12), 140.3 (s, C-13), 138.8 (s, C-17) and 19.0 (q, C-30), instead of *δ*_C_ 135.7 (d, C-8), 134.2 (s, C-8), 33.3 (t, C-11), 29.3 (d, C-12), 143.8 (s, C-13), 134.6 (s, C-17) and 21.5 (q, C-30) in kadindutic acid, indicating compound **2** was generated from kadindutic acid for the double bond moved from C-8-C-9 to C-11-C-12, and the methyl group 30-Me moved from C-12 to C-8. Furthermore, the determined molecular formula C_15_H_22_O_4_ and the key ^1^H-^1^H COSY correlations with H-9 [*δ*_H_ 2.40 (1H, m)]/H-11 [*δ*_H_ 6.27 (1H, dd, *J* = 2.9, 10.2 Hz)], H-11/H-12 [*δ*_H_ 5.59 (1H, d, *J* = 10.2 Hz)] and the HMBC correlations from H-30 [*δ*_H_ 0.94 (1H, m)] to C-8 (*δ*_C_ 37.5 s), C-9 (*δ*_C_ 46.9 d) and C-14 (*δ*_C_ 55.1 s), from H-11to C-9 and C-10 (*δ*_C_ 35.6 s) and from H-12 to C-13 (*δ*_C_ 140.3 s) confirmed this hypothesis. The other ^1^H-^1^HCOSY and HMBC correlations (Figure 2) also confirmed this atom connectivity. The relative configuration of compound **1** was determined to be closed to the kadindutic acid (Figure 2) [12] except for the β-oriented of C-30, and *α*-oriented of H-9 and C-18 by ROESY cross-peaks H-19 [*δ*_H_ 0.89 (3H, s)]/H-30 [*δ*_H_ 0.95 (3H, s)], H-9/H-18 [*δ*_H_ 1.02 (3H, s)]. Thus, the structure of compound **1** was assigned as shown and named kadsuricoccins B.

Compounds **1–5** were all tested for their AchE-inhibitory activity by Ellman’s Method as described before [13]. As a result, compounds **1–3** (at the concentration of 94 nM) showed moderate activity with the AChE inhibit index (AII) 68.96 ± 0.19, 57.8 ± 0.11 and 37.55 ± 0.12 % at 94 nM, respectively, compared with the negative control (AII 8.94 ± 0.09%) and positive control (tacrine with AII 79.8 ± 0.20 %, 9.4 nM) (Table 2). The LD_50_ of the compounds **1** and **2** were predicted as 4,000 mg/kg and 900 mg/kg, with toxicity categorized into classes 5 and 4, respectively. Other ADMET properties were also predicted and analyzed (see supporting information).

## 3. Experimental

### 3.1. General

Optical rotations were measured on an MCP 5100 polarimeter (Anton Paar, Graz, Austria), UV spectra were acquired on a UV-250 spectrometer (Shimadzu, Kyoto, Japan) and IR spectra were measured on a Nicolet 380 spectrometer (Thermo, Berkeley, CA, USA) with KBr pellets. 1D and 2D NMR spectra were obtained using an AV-500 instrument (Bruker, Billerica, MA, USA) with TMS as an internal standard, and ESIMS and HRESIMS were recorded with an Autospec Premier spectrometer (Waters, Milford, MA, USA) or a Micromass Autospec-Uitima-TOF (Waters, Milford, MA, USA). The column chromatography (CC) was performed on ODS (40–70 μM, Fuji Silysia Chemical Ltd., Nagoya, Japan), Silica gel (200–300 mesh, Qingdao Marine Chemical Inc., Qingdao, China), and Sephadex LH-20 (GE Healthcare Bio-Sciences AB, Uppsala, Sweden). Fractions were color detected by TLC and heating after being soaked with 10% H_2_SO_4_ in ethanol (EtOH). 

The toxicity and other ADMET properties (Druglikeness, Pharmacokinetics, Lipophilicity, etc.) of the compounds were also analyzed using the SwissADME (http://www.swissadme.ch/index.php) and ProTox-II—Prediction of Toxicity of Chemicals (http://tox.charite.de/protox_II/).

### 3.2. Plant Material

The stems of Heilaohu [*Kadsura coccinea* (Lem.) A. C. Smith.] were collected in Changjiang Hainan Province (E 109° 10′ 0.53′′, N 19° 3′ 39.7′′), People′s Republic of China, in May 2017. The voucher specimen (HUANG00012) identified by Prof. Dr. Jun Wang (Institute of Tropical Bioscience and Biotechnology, Chinese Academy of Tropical Agriculture Sciences) was deposited at the Hainan Key Laboratory for Research and Development of Natural Products from Li Folk Medicine, Institute of Tropical Bioscience and Biotechnology, Chinese Academy of Tropical Agriculture Sciences, Haikou, People’s Republic of China. 

### 3.3. Extraction and Isolation

The air-dried roots of *K. coccinea* (35 kg) were powdered and extracted with 95% EtOH for two weeks (3 × 100 L). The combined EtOH solution was concentrated with a rotary evaporator and followed by suspension in 2.5 L of water, then extracted successively with petroleum ether extract (3 × 5 L). The petroleum ether extract (305 g) was first subjected to silica gel (*φ* 16 × 150 cm) CC eluted with petroleum ether/ethyl acetate (EtOAc) (from 50:1 to 1:1, *v*/*v*) to obtain fractions A–C. Fraction B (36) was chromatographed repeatedly over an MCI with MeOH and acetone, an ODS (*φ* 4 × 18 cm) with MeOH/H_2_O (gradient elution with 30, 40, 50, 60, 70, 80 and 90%, each 500 mL), and Sephadex LH-20 CC (MeOH/CHCl_3_ 1:1 as solvent) to yield cycloartenone (**4**) (2.0 mg), and 24-methyl-8-lanoten-3-one (**5**) (3.0 mg), respectively. Fraction C (15 g) was then subjected to CC over silica gel (*φ* 6 × 45 cm) eluted with petroleum ether/acetone (from 5:1 to 2:1, *v*/*v*) to give four fractions C1–C3. Fractions C1 (1.5 g) was chromatographed repeatedly over an ODS (*φ* 4 × 18 cm) with MeOH/H_2_O (gradient elution with 30, 40, 50, 60, 70, 80 and 90%, each 500 mL) and Sephadex LH-20 CC (MeOH/CHCl_3_ 1:1 as solvent) to yield auranticanol C (**4**) (1.5 mg). Fraction C2 (5.6) was chromatographed repeatedly over an ODS (*φ* 4 × 18 cm) with MeOH/H_2_O (gradient elution with 20, 30, 40, 50, 60, 70, 80 and 90%, each 500 mL) to obtain fractions C2a–C2d. Fractions C2a–C2d were chromatographed repeatedly over Sephadex LH-20 CC, using MeOH/CHCl_3_ 1:1 as solvent to yield **1** (1.9 mg), **2** (7.0 mg) and **3** (3.8 mg), respectively.

#### 3.3.1. Kadsuricoccin A (**1**)

Colorless, amorphous powder; [α${]}_{D}^{18.2}$ +23.8 (*c* 0.02, MeOH); UV (MeOH) *λ*_max_ (logε) 294 (2.04), 255 (2.36), 215 (2.23), 209 (1.68) nm; IR (KBr) *ν*_max_ 2963, 2935, 2924, 1712, 1691, 1623, 1344, 1321, 1302; ^1^H and ^13^C-NMR data see Table 1; ESIMS positive *m*/*z* [M + Na]^+^ 381(50); HRESIMS *m*/*z* [M + Na]^+^ 381.2503 (calcd for C_23_H_34_O_3_Na, 381.2508).

#### 3.3.2. Kadsuricoccin B (**2**)

Colorless oil; [α${]}_{D}^{17.0}$ −6.14 (*c* 0.10, CHCl_3_); UV (MeOH) *λ*_max_ (logε) 286 (0.59), 254 (2.10), 218 (2.09) nm; IR (KBr) *ν*_max_ 3388, 2958, 1705, 1457, 1380, 1243, 1099, 1027, 798 cm^−1^; ^1^H and ^13^C-NMR data see Table 1; ESIMS positive *m*/*z* [M + Na]^+^ 475 (40); HRESIMS *m*/*z* [M + Na]^+^ 475.3208 (calcd for C_30_H_44_O_3_Na^+^, 475.3183).

### 3.4. Acetylcholinesterase (AchE)-Inhibitory Bioassay

The AchE-inhibitory activity of the compounds was tested as described by Ellman’s Method in the literature [13,14]. The mixed reaction solution contained test compound soln. (100 μM in dimethyl sulfoxide (DMSO), 10 μL, tacrine as a positive control, 10 μM in DMSO, 10 μL,), phosphate buffer (pH 8.0 (slightly alkali), 1010 μL), and AchE soln. (0.04 U/100 μL, 40 μL). After being incubated for 20 min (at 30 °C), the reaction was initiated by the addition of DTNB [5,5′-dithiobis (2-nitrobenzoic acid), 6.25 mM] 20 μL and acetylthiocholine 20 μL. 

The acetylthiocholine hydrolysis reaction was monitored at 405 nm after 30 min later. All the reactions were performed in triplicate. The AChE inhibit index (AII) was calculated according to the equation: %inhibition = (*E* − *S*)/*E* × 100 (*S*, the activity of enzyme with test compound; *E*, activity of the enzyme without test compound). 

## 4. Conclusions

The skeleton diversity of triterpenoids in the *Schisandraceae* family makes the phytochemisty and organic synthesis chemistry of this family of great interest to our research field [5,15]. An octanordammarane (a kind of octanary nor-triterpenoid) isolated from this genus firstly showed its accessibility from the *Schisandraceae* plant, whilst other compounds isolated from *Kadsura coccinea* indicated the probability of biosynthesis as other *Kadsura* plants [16]. Furthermore, the bioactivity evaluation assay showed that kadsuricoccins A and B have moderate AChE inhibitory activities with predicted lower toxicity as some pentacyclic triterpenoids [17], while the AChE was an important target for Alzheimer’s Disease (AD) [18] and myasthenia gravis [19]. With that, these two new compounds were found that they may be used as a health supplement.

## Figures and Tables

**Table 1:** ^1^H (500 MHz) and ^13^C nuclear magnetic resonance (NMR) (125 MHz) Data of Compounds **1**–**2**.

Compound	1 (in CDCl_3_)		2 (in CDCl_3_)	
No.	*δ*_H_ mult. (*J* in Hz)	*δ*_C_	*δ*_H_ mult. (*J* in Hz)	*δ*_C_
**1**	2.01 m 1.87 m	30.2 t	1.71 m 2.21 m	32.1 t
**2**	2.29 m	30.3 t	2.87 ddd, (7.2, 12.0, 14.8) 2.21 m	33.2 t
**3**	-	174.8 s	-	221.6 s
**4**	-	147.0 s	-	47.0 s
**5**	2.18 m	47.0 d	2.41 s	46.3 d
**6**	1.95 m 1.49 m	22.1 t	1.53 dd, (7.6, 12.9) 1.34 m	19.0 t
**7**	2.08 m 1.17 m	27.3 t	1.97 m 1.29 d, (6.3)	31.2 t
**8**	-	37.4 s	-	37.5 s
**9**	2.25 m	41.2 d	2.40 m	46.9 d
**10**		37.7 s		35.6 s
**11**	2.21 m	24.7 t	6.27 dd, (2.9, 10.2)	121.5 d
**12**	6.65 dd, (3.4, 3.6)	131.4 d	5.59 d, (10.2)	126.6 d
**13**	-	144.1 s	-	140.3 s
**14**	-	48.6 s	-	55.1 s
**15**	1.99 m 1.54 m	26.8 t	2.43 m	27.8 t
**16**	2.46 m 2.28 m	36.1 t	1.38 m	28.6 t
**17**	-	207.1 s	-	138.8 s
**18**	1.18 s	23.4 q	1.02 s	22.0 q
**19**	0.91 s	17.7 q	0.89 s	23.9 q
**20**	4.96 s 4.84 s	113.4 t	2.66 m	31.7 d
**21**	1.82 s	26.9 q	1.01 d, (7.6)	19.2 q
**22**	0.94 s	25.1 q	1.44 m	34.9 t
**23/OMe**	3.69 s	51.7 q	2.32 dd, (8.8, 16.5)	28.1 t
**24**			5.86 t, (7.2)	139.3 d
**25**				128.7 s
**26**				171.5 s
**27**			1.87 s	19.7 q
**28**			1.11 s	28.2 q
**29**			1.03 s	18.3 q
**30**			0.95 s	19.0 q

**Table 2:** The acetylcholinesterase (AChE) inhibit index (AII) of compounds **1**–**5** (94 nM) with the inhibition rate of this same acetylcholinesterase.

Compounds	AII (%)
**1**	68.96 ± 0.19
**2**	57.8 ± 0.11
**3**	37.75 ± 0.12
**4**	17.23 ± 0.08
**5**	25.66 ± 0.18
**Blank control**	8.94 ± 0.09
**Tacrine (positive control, 9.4 nM)**	79.80 ± 0.20

## Supplementary Materials

The following are available online.
