# Supplementary material for: Two New AChE Inhibitors Isolated from Li Folk Herb Heilaohu “Kadsura coccinea” Stems
Source: Molecules. 2019 Oct 8;24(19):3628. doi: 10.3390/molecules24193628 (PMC6804138; doi:10.3390/molecules24193628)
Supplement: Supplementary file 1 [file molecules-24-03628-s001.pdf]

Supporting information to

Two New AChE Inhibitors Isolated from Li Folk Herb Heilaohu "*Kadsura coccinea*" stems.

Sheng Zhuo Huang[a,#], Lin Ping Duan[b,#], Hao Wang[a], Wen Li Mei,[a,] * and Hao Fu Dai[a,]*

Hainan Key Laboratory for Research and Development of Natural Products from Li Folk Medicine, Ministry of Agriculture, Institute of Tropical Bioscience and Biotechnology, Chinese Academy of Tropical Agriculture Sciences, Haikou 571101, College of Food Science and technology, Nanjing Agricultural University, Nanjing 210095

* To whom correspondence should be addressed. Tel:. Fax: +86-0898-66968036.

E-mail:daihaofu@itbb.org.cn, meiwenli@itbb.org.cn

1.^{1}H NMR spectrum of kadsuricoccin A (**1**)........................2
2. ^{13}C NMR and DEPT spectrum of kadsuricoccin A (**1**)........................2
3. HSQC spectrum of kadsuricoccin A (**1**)........................3
4. ^{1}H-^{1}H COSY spectrum of kadsuricoccin A (**1**)........................3
5. HMBC spectrum of kadsuricoccin A (**1**)........................4
6. ROESY spectrum of kadsuricoccin A (**1**)........................4
7.^{1}H NMR spectrum of kadsuricoccin B (**2**)........................5
8. ^{13}C NMR and DEPT spectrum of kadsuricoccin B (**2**)........................5
9. HSQC spectrum of kadsuricoccin B (**2**)........................6
10. ^{1}H-^{1}H COSY spectrum of kadsuricoccin B (**2**)........................6
11. HMBC spectrum of kadsuricoccin B (**2**)........................7
12. ROESY spectrum of kadsuricoccin B (**2**)........................7
13. calculated properties of kadsuricoccins A and B (**1**, **2**)........................8

1.^{1}H NMR spectrum of kadsuricoccin A (**1**)

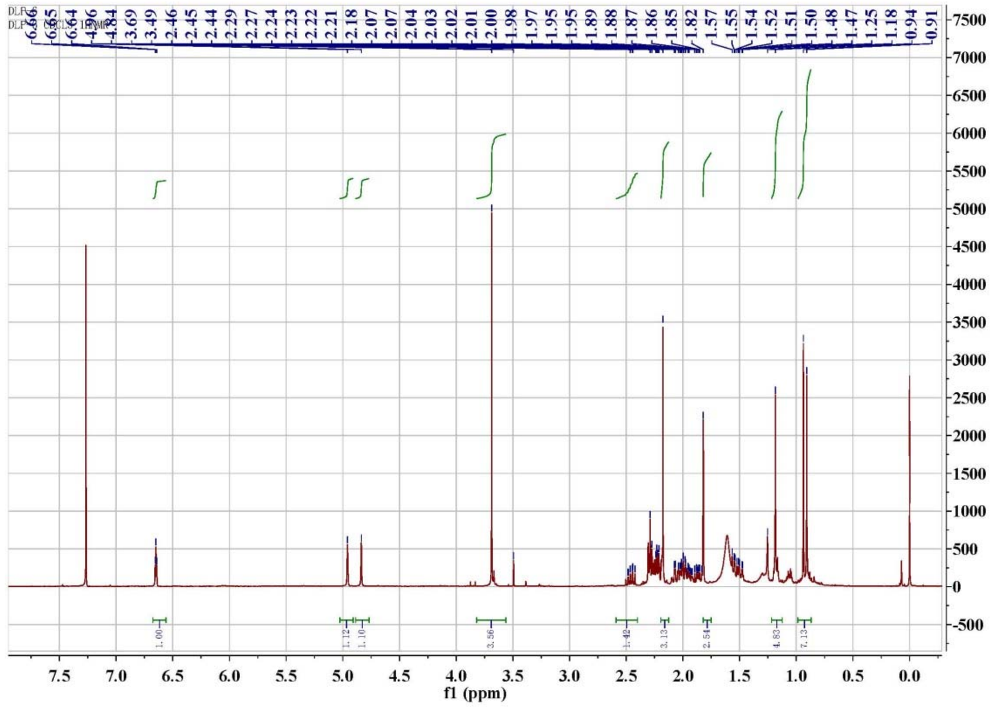

2. ^{13}C NMR and DEPT spectrum of kadsuricoccin A (**1**)

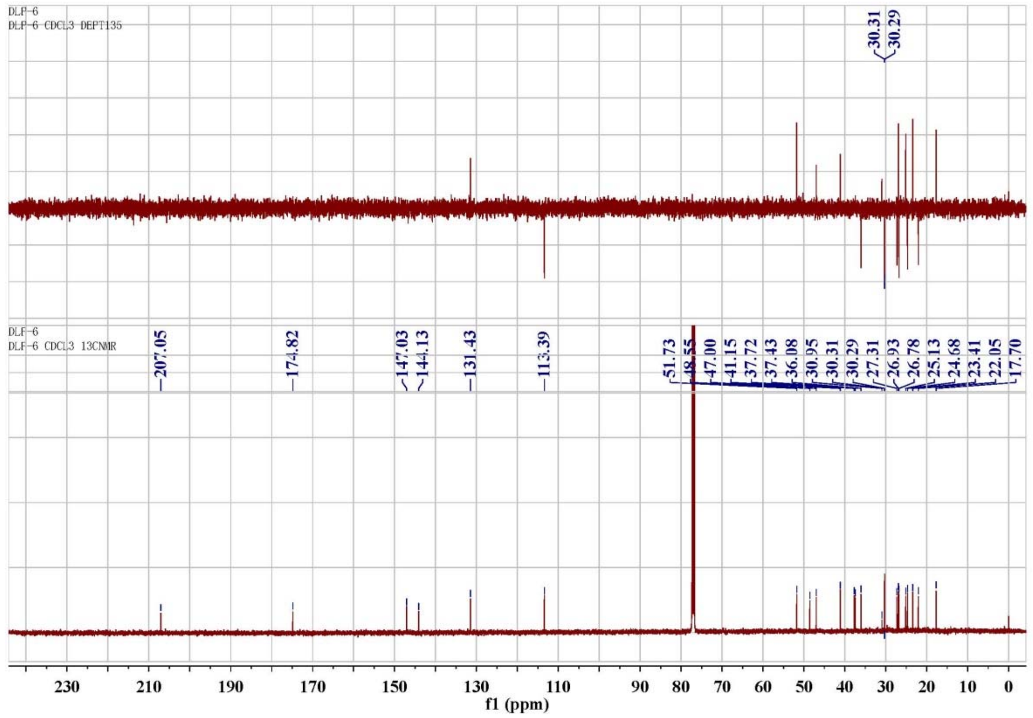

3. HSQC spectrum of kadsuricoccin A (**1**)

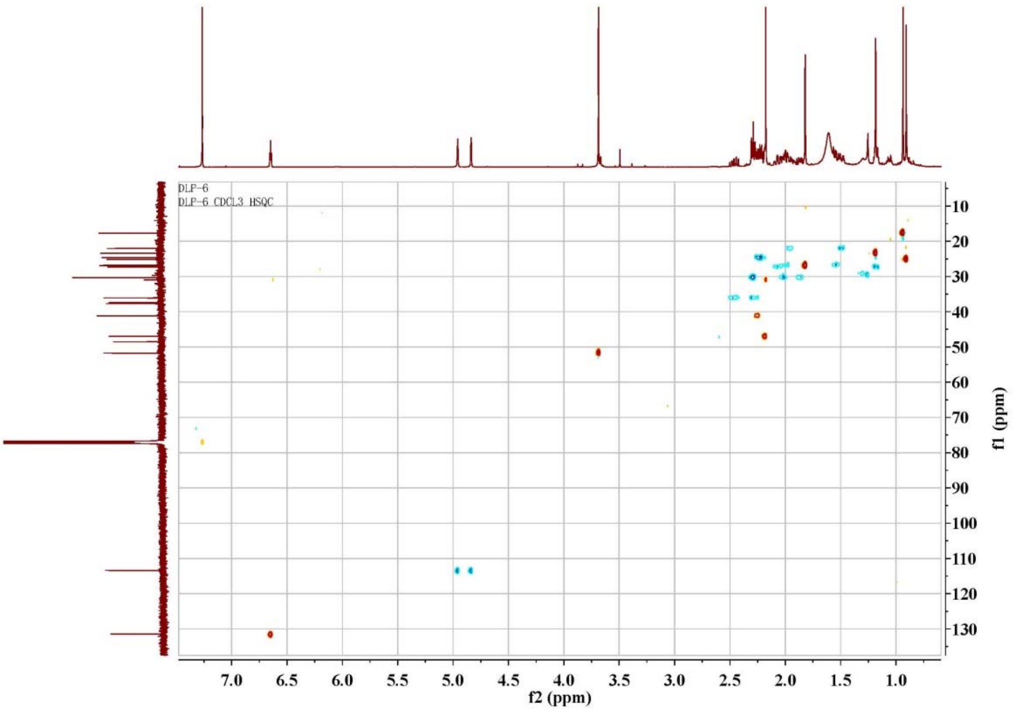

4. ^{1}H-^{1}H COSY spectrum of kadsuricoccin A (**1**)

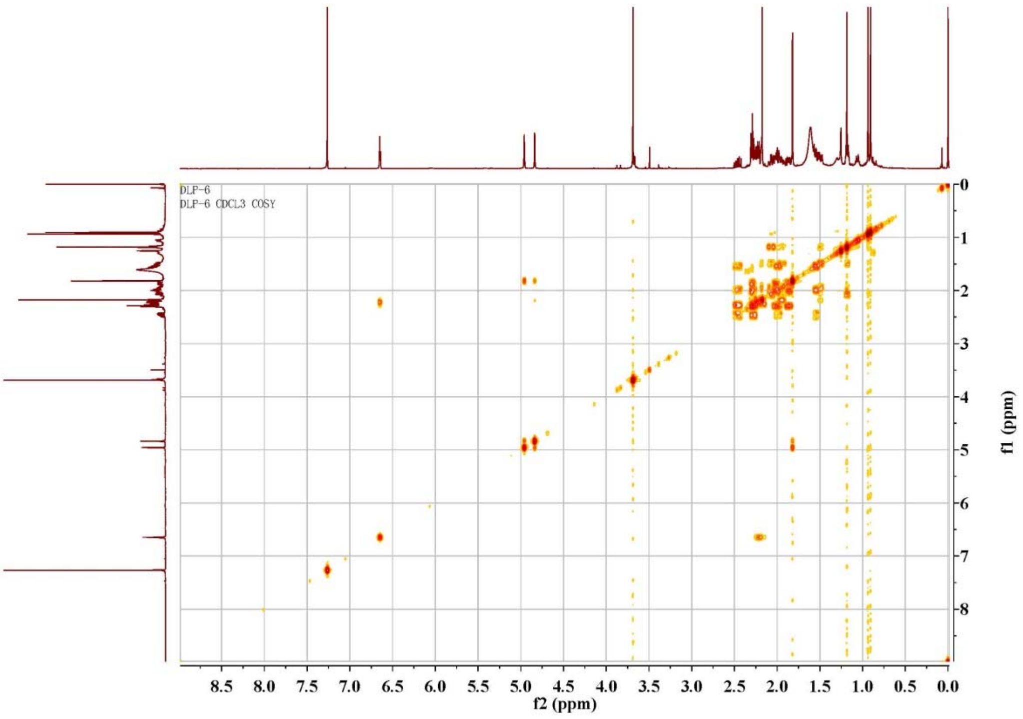

5. HMBC spectrum of kadsuricoccin A (**1**)

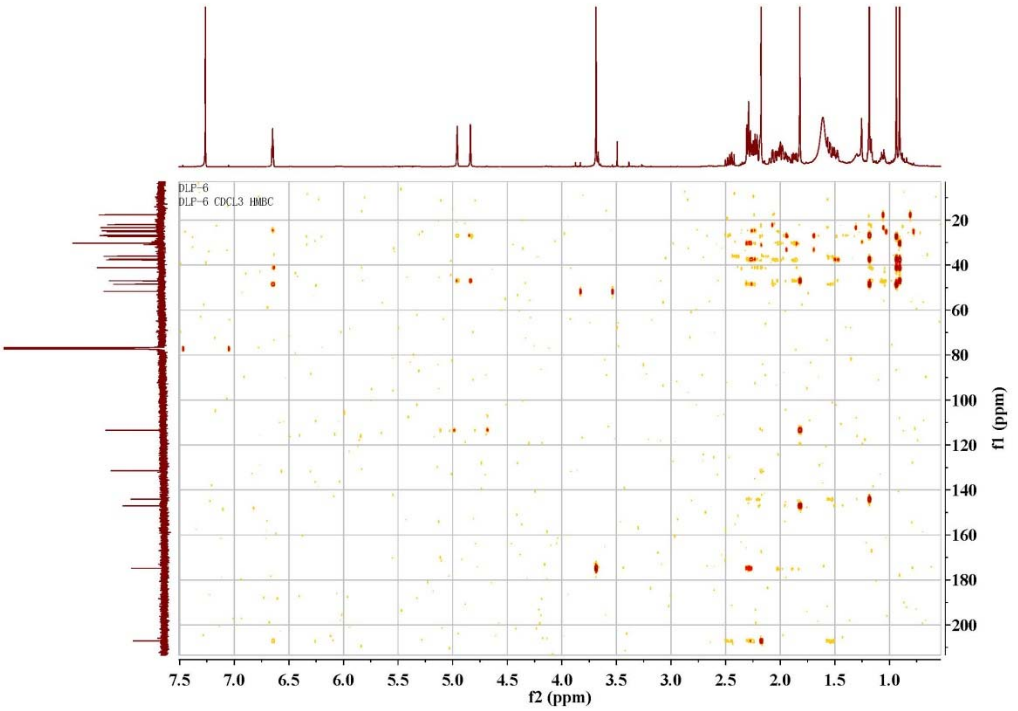

6. ROESY spectrum of kadsuricoccin A (**1**)

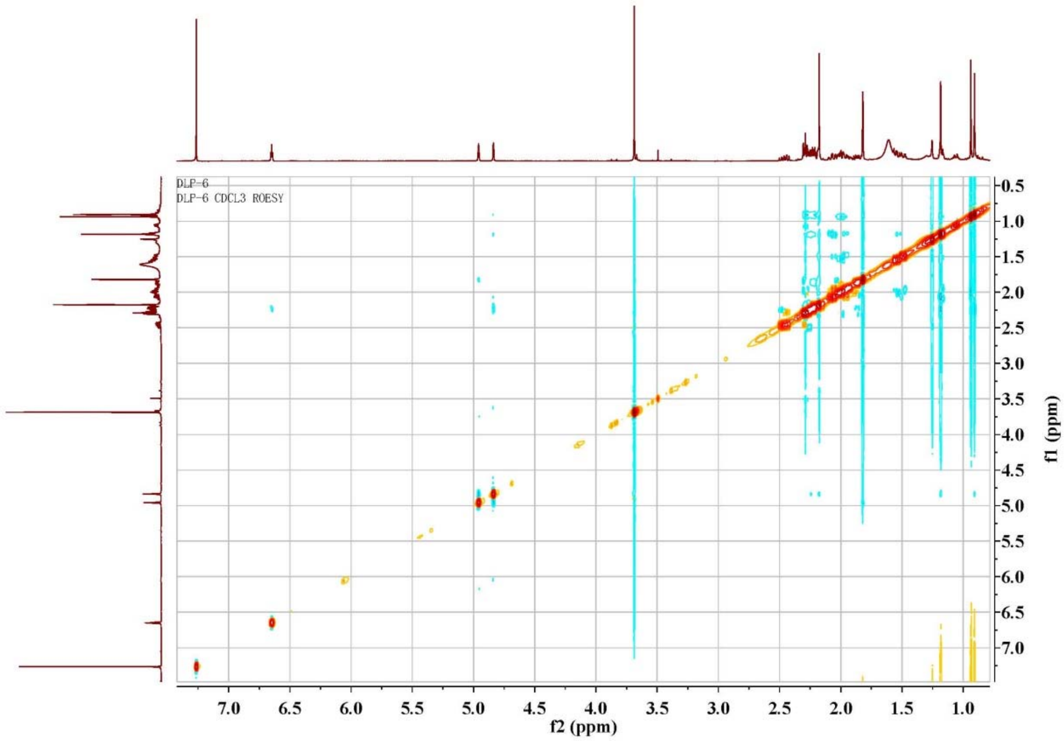

7.^{1}H NMR spectrum of kadsuricoccin B (**2**)

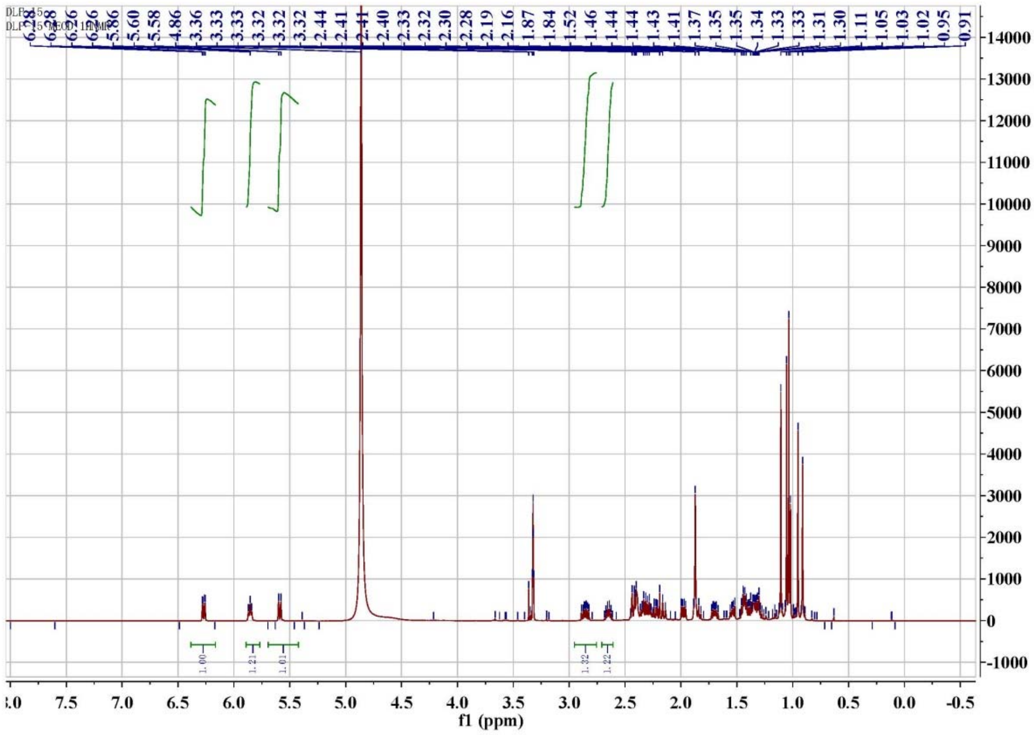

8. ^{13}C NMR and DEPT spectrum of kadsuricoccin B (**2**)

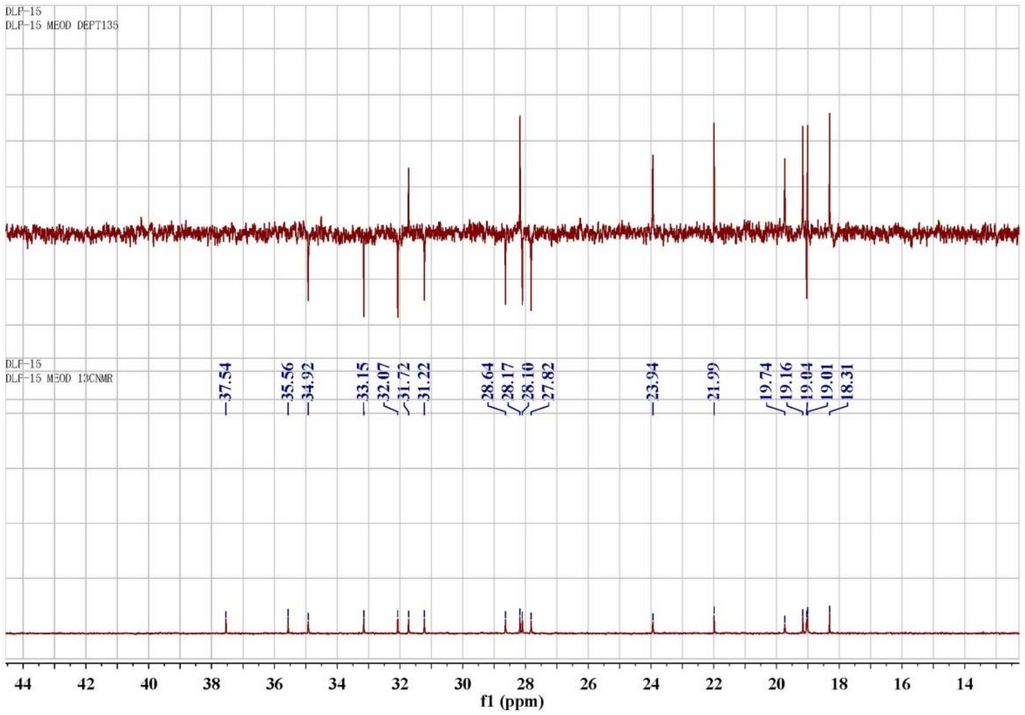

9. HSQC spectrum of kadsuricoccin B (**2**)

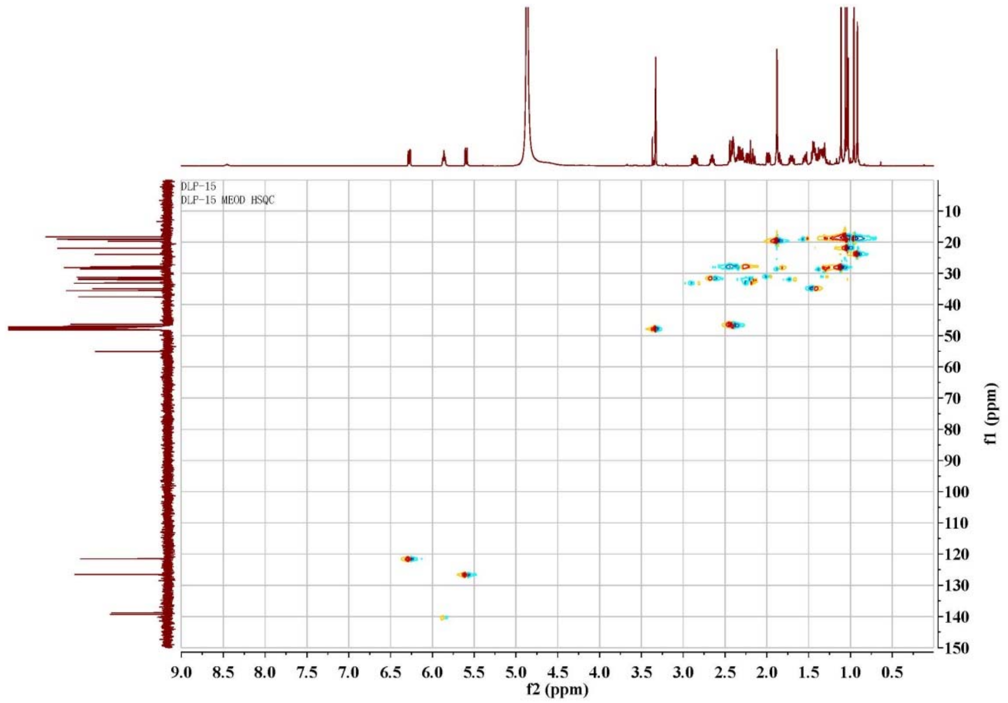

10. ^{1}H-^{1}H COSY spectrum of kadsuricoccin B (**2**)

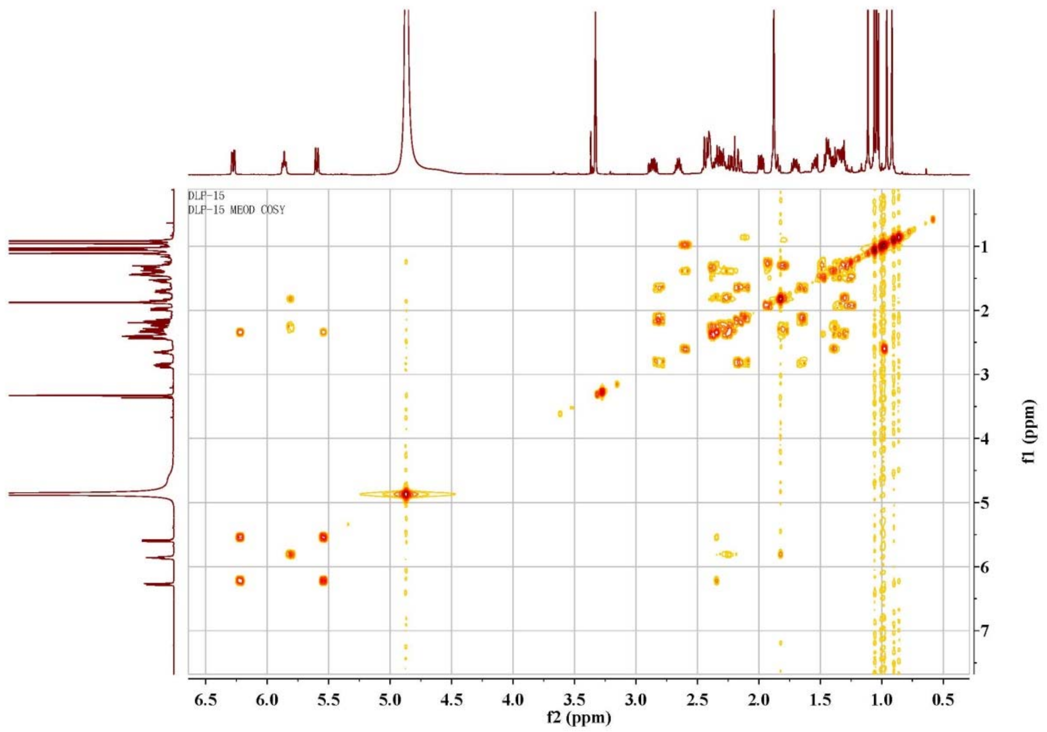

11. HMBC spectrum of kadsuricoccin B (**2**)

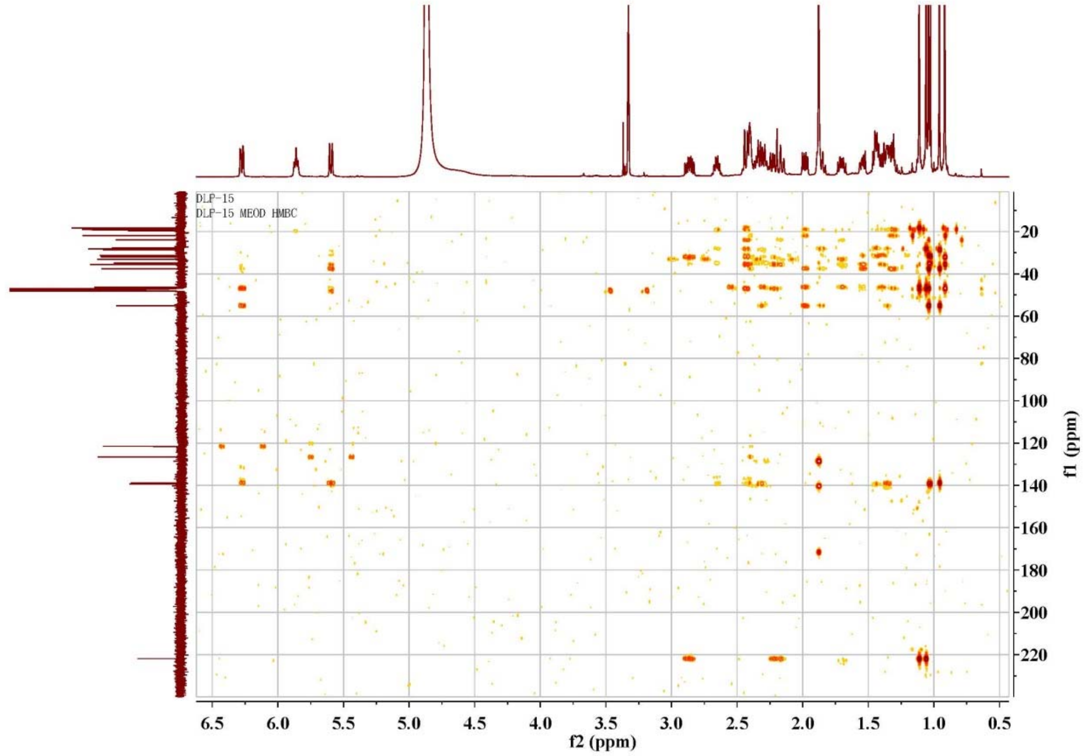

12. ROESY spectrum of kadsuricoccin B (**2**)

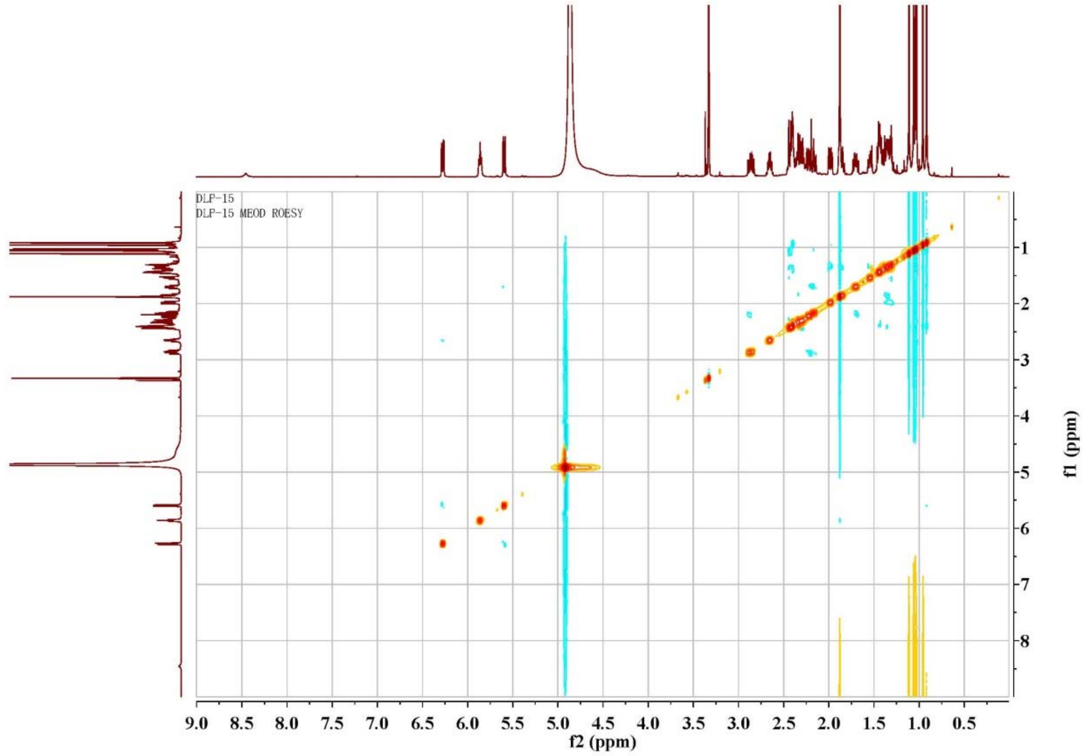

13. calculated properties of kadsuricoccins A and B (**1**, **2**)

Molecule 1

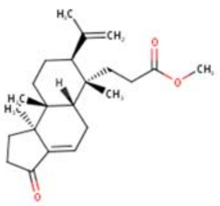

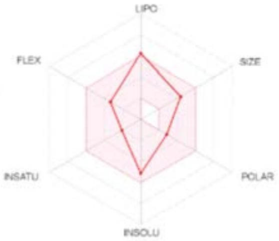

SMILES: COC(=O)CC[C@@]1(C)[C@@H](CC[C@@]2([C@H]1CC=C1[C@@]2(C)CCC1=O)C)C(=C)C

Physicochemical Properties	
Formula	C23H34O3
Molecular weight	358.51 g/mol
Num. heavy atoms	26
Num. arom. heavy atoms	0
Fraction Csp3	0.74
Num. rotatable bonds	5
Num. H-bond acceptors	3
Num. H-bond donors	0
Molar Refractivity	106.09
TPSA	43.37 Å²

Lipophilicity	
Log $P_{o/w}$ (iLOGP)	3.69
Log $P_{o/w}$ (XLOGP3)	5.40
Log $P_{o/w}$ (WLOGP)	5.25
Log $P_{o/w}$ (MLOGP)	4.22
Log $P_{o/w}$ (SILICOS-IT)	5.47
Consensus Log $P_{o/w}$	4.81

Water Solubility	
Log *S* (ESOL)	-5.13
Solubility	2.63e-03 mg/ml ; 7.33e-06 mol/l
Class	Moderately soluble
Log *S* (Ali)	-6.07
Solubility	3.08e-04 mg/ml ; 8.60e-07 mol/l
Class	Poorly soluble
Log *S* (SILICOS-IT)	-5.47
Solubility	1.22e-03 mg/ml ; 3.41e-06 mol/l
Class	Moderately soluble

Pharmacokinetics	
GI absorption	High
BBB permeant	Yes
P-gp substrate	No
CYP1A2 inhibitor	No
CYP2C19 inhibitor	Yes
CYP2C9 inhibitor	Yes
CYP2D6 inhibitor	Yes
CYP3A4 inhibitor	Yes
Log K_p (skin permeation)	-4.65 cm/s

Druglikeness	
Lipinski	Yes; 1 violation: MLOGP>4.15
Ghose	Yes
Veber	Yes
Egan	Yes
Muegge	No; 1 violation: XLOGP3>5
Bioavailability Score	0.55

Medicinal Chemistry	
PAINS	0 alert
Brenk	1 alert: isolated_alkene
Leadlikeness	No; 2 violations: MW>350, XLOGP3>3.5
Synthetic accessibility	4.85

Oral toxicity prediction results for input compound

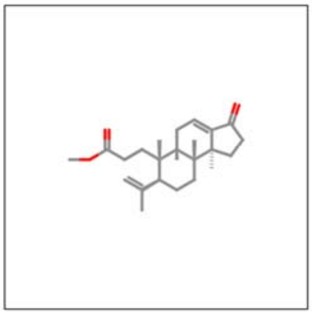

Predicted LD50: 4000mg/kg

Predicted Toxicity Class: 5

1 2 3 4 5 6

Average similarity: 88.35%

Prediction accuracy: 70.97%

Name	User defined
Molweight	358.51
Number of hydrogen bond acceptors	3
Number of hydrogen bond donors	0
Number of atoms	28
Number of bonds	30
Number of rings	3
Number of rotatable bonds	5
Total charge	0
Molecular Polar Surface Area	43.37

Comparison of input compound with dataset compounds

Molecule 1

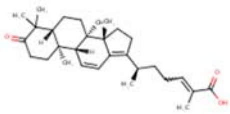

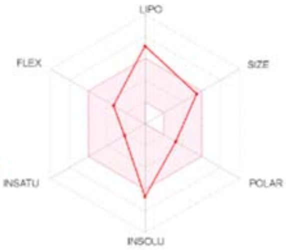

SMILES OC(=O)/C(=C\CC[C@H](C1=C2C=C[C@H]3[C@@]([C@@]2(CC1)C)(C)CC[C@@H]1[C@]3(C)CCC(=O)C1(C)C)C)/C

Physicochemical Properties	
Formula	C30H44O3
Molecular weight	452.67 g/mol
Num. heavy atoms	33
Num. arom. heavy atoms	0
Fraction Csp3	0.73
Num. rotatable bonds	5
Num. H-bond acceptors	3
Num. H-bond donors	1
Molar Refractivity	137.38
TPSA	54.37 Å²

Lipophilicity	
Log $P_{o/w}$ (iLOGP)	4.06
Log $P_{o/w}$ (XLOGP3)	6.92
Log $P_{o/w}$ (WLOGP)	7.53
Log $P_{o/w}$ (MLOGP)	5.55
Log $P_{o/w}$ (SILICOS-IT)	6.92
Consensus Log $P_{o/w}$	6.20

Water Solubility	
Log *S* (ESOL)	-6.68
Solubility	9.54e-05 mg/ml ; 2.11e-07 mol/l
Class	Poorly soluble
Log *S* (Ali)	-7.87
Solubility	6.05e-06 mg/ml ; 1.34e-08 mol/l
Class	Poorly soluble
Log *S* (SILICOS-IT)	-6.38
Solubility	1.87e-04 mg/ml ; 4.13e-07 mol/l
Class	Poorly soluble

Pharmacokinetics	
GI absorption	Low
BBB permeant	No
P-gp substrate	Yes
CYP1A2 inhibitor	No
CYP2C19 inhibitor	No
CYP2C9 inhibitor	Yes
CYP2D6 inhibitor	No
CYP3A4 inhibitor	No
Log K_p (skin permeation)	-4.15 cm/s

Druglikeness	
Lipinski	Yes; 1 violation: MLOGP>4.15
Ghose	No; 3 violations: WLOGP>5.6, MR>130, #atoms>70
Veber	Yes
Egan	No; 1 violation: WLOGP>5.88
Muegge	No; 1 violation: XLOGP3>5
Bioavailability Score	0.56

Medicinal Chemistry	
PAINS	0 alert
Brenk	1 alert: michael_acceptor_1
Leadlikeness	No; 2 violations: MW>350, XLOGP3>3.5
Synthetic accessibility	6.25

Oral toxicity prediction results for input compound

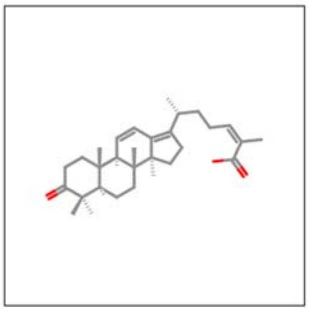

Predicted LD50: 900mg/kg

Predicted Toxicity Class: 4

1 2 3 4 5 6

Average similarity: 73.35%

Prediction accuracy: 69.26%

Name	User defined
Molweight	452.67
Number of hydrogen bond acceptors	3
Number of hydrogen bond donors	0
Number of atoms	36
Number of bonds	39
Number of rings	4
Number of rotable bonds	5
Total charge	0
Molecular Polar Surface Area	54.37

Comparison of input compound with dataset compounds
